# Poor ability to resist tempting calorie rich food is linked to altered balance between neural systems involved in urge and self-control

**DOI:** 10.1186/1475-2891-13-92

**Published:** 2014-09-16

**Authors:** Qinghua He, Lin Xiao, Gui Xue, Savio Wong, Susan L Ames, Susan M Schembre, Antoine Bechara

**Affiliations:** Faculty of Psychology, Southwest University, Beibei, Chongqing, China; Brain and Creativity Institute and Department of Psychology, University of Southern California, Los Angeles, CA USA; National Key Laboratory of Cognitive Neuroscience and Learning & IDG/McGovern Institute for Brain Research, Beijing Normal University, Beijing China; Department of Special Education and Counselling, The Hong Kong Institute of Education, Hong Kong, China; School of Community and Global Health, Claremont Graduate University, Claremont, CA USA; Department of Behavioral Science, The University of Texas MD Anderson Cancer Center, Houston, TX USA

**Keywords:** Decision making, Obesity, fMRI, Food, Habitual system, Prefrontal system

## Abstract

**Background:**

The loss of self-control or inability to resist tempting/rewarding foods, and the development of less healthful eating habits may be explained by three key neural systems: (1) a hyper-functioning striatum system driven by external rewarding cues; (2) a hypo-functioning decision-making and impulse control system; and (3) an altered insula system involved in the translation of homeostatic and interoceptive signals into self-awareness and what may be subjectively experienced as a feeling.

**Methods:**

The present study examined the activity within two of these neural systems when subjects were exposed to images of high-calorie versus low-calorie foods using functional magnetic resonance imaging (fMRI), and related this activity to dietary intake, assessed by 24-hour recall. Thirty youth (mean BMI = 23.1 kg/m^2^, range = 19.1 - 33.7; age =19.7 years, range = 14 - 22) were scanned using fMRI while performing food-specific go/nogo tasks.

**Results:**

Behaviorally, participants more readily pressed a response button when go trials consisted of high-calorie food cues (HGo task) and less readily pressed the response button when go trials consisted of low-calorie food cues (LGo task). This habitual response to high-calorie food cues was greater for individuals with higher BMI and individuals who reportedly consume more high-calorie foods. Response inhibition to the high-calorie food cues was most difficult for individuals with a higher BMI and individuals who reportedly consume more high-calorie foods. fMRI results confirmed our hypotheses that (1) the "habitual" system (right striatum) was more activated in response to high-calorie food cues during the go trials than low-calorie food go trials, and its activity correlated with participants’ BMI, as well as their consumption of high-calorie foods; (2) the prefrontal system was more active in nogo trials than go trials, and this activity was inversely correlated with BMI and high-calorie food consumption.

**Conclusions:**

Using a cross-sectional design, our findings help increase understanding of the neural basis of one’s loss of ability to self-control when faced with tempting food cues. Though the design does not permit inferences regarding whether the inhibitory control deficits and hyper-responsivity of reward regions are individual vulnerability factors for overeating, or the results of habitual overeating.

**Electronic supplementary material:**

The online version of this article (doi:10.1186/1475-2891-13-92) contains supplementary material, which is available to authorized users.

## Background

Overweight and obesity, expressed as above-normal body mass index (BMI > 24.9 kg/m^2^), is a public health challenge worldwide. In the United States, nearly 70% of adults are overweight or obese [1]. Overweight and obesity are associated with increased risk for cardiovascular/metabolic diseases, as well as some cancers [2]. Although there is no clear explanation of the primary cause of overweight and obesity, excessive weight gain is the known result of chronic positive energy imbalances favoring calories consumed over calories expended [3]. Thus, it is important to identify underlying mechanisms that relate to behaviors associated with excessive energy intake in order to address the obesity epidemic. Much research to date has explored the underlying influences of genetic, hormonal, and metabolic mechanisms related to obesity and dietary intake [4–9]. Fewer studies have sought to understand the underlying, and potentially modifiable, neural mechanisms that motivate decisions about "what" and "how much" to eat [10–19].

Mounting evidence suggests that the difficulty to resist highly palatable, calorie-rich foods represents a special case of addictive behavior with similarities to other addictions [20–24]. Several studies have shown that individuals who eat excessively are unable to make optimal food-related choices [25, 26], characterized by a tendency to choose the immediate reward of a food item at the expense of potentially long-term negative consequences [27]. Based on these findings, we hypothesized that a loss of self-control or inability to resist tempting/rewarding foods, and the development of less healthful eating habits (e.g., greater intake of high-calorie foods), may be explained by three key neural systems: (1) a hyper-functioning striatum driven by external rewarding cues, including highly rewarding foods. We have referred to this neural system as the "impulsive" system [28–31]. This is the same system that has been shown, in animal models, to be responsible for the development of automatic and habitual behaviors in response to reward cues [32, 33]; (2) a hypo-functioning decision-making and impulse control system, which includes the mesial orbitofrontal cortex, the sub-genual regions of the anterior cingulate cortex (ACC), and adjacent areas within the ventral medial prefrontal cortex. In addition, inhibition control and response inhibition have also been linked to lateral regions of the prefrontal cortex [34–39]. We have referred to this neural system as the "reflective" system or prefrontal system [28]; and (3) based on more recent evidence on the effects of brain lesions on smoking behavior [40], we hypothesized that the strength of the two previously outlined neural systems can be modulated by the insula system involved in urge and craving, which includes the anterior insula. Indeed, the anterior insula is thought to be involved in the translation of homeostatic and interoceptive signals into self-awareness of subjective feelings [41–45]. Accordingly, we have proposed that interoceptive signals triggered by food deprivation, or by exposure to food cues, are relayed to the insular cortex and translated into craving and what may become subjectively experienced as an intense urge to eat. Consistent with this conceptualization, neuroimaging studies have shown the impulsive system (striatum) to be consistently more active during exposure to high-calorie foods when compared to low-calorie foods or control images [13, 16, 46–55]. This effect is greater for overweight versus normal weight participants [47, 53, 56, 57], and could potentially predict short-and long-term outcomes in weight-loss programs [58]. On the other hand, an increasing number of studies suggest that activity within the prefrontal system is also altered in response to food cues [59, 60]. These results are consistent with the idea that these systems, which were previously shown to have altered activity in cases of other substance addictions, also show some altered responses to visual food cues in obese individuals [46, 61–65].

The present study used functional magnetic resonance imaging (fMRI) techniques to investigate brain activity related to the neural systems described above during food-specific go/nogo tasks consisting of high- and low-calorie food cues. Specifically, we tested hypotheses related to the activities of the impulsive (striatum) and reflective (prefrontal) neural systems in response to images of high-calorie versus low-calorie food cues, and related these activities to dietary intake. Participants were adolescents and young adults who represent an intriguing group to study given the relatively delayed maturation of the prefrontal cortex [66–68], and, as a result, the potential for making disadvantageous food choices. Indeed, this population has a tendency to make less healthful food choices [69], and such choices are facilitated by school environments and university campuses where accessibility to highly palatable, high-calorie food options is relatively unrestricted.

## Methods

### Participants

Thirty (17 female) healthy adolescents and young adults aged 19.7 years (SD = 1.7, range = 14 - 22) years were recruited (see Table 1). Their average BMI was 23.1 kg/m^2^ (SD = 3.0, range = 19.1 - 33.7). None of the participants were currently receiving clinical treatment for obesity. All participants had normal or corrected-to-normal vision. To rule out participants with certain neuropsychiatric disorders, medications, or health issues that could impact the neuroimaging results, we used (1) the Structured Clinical Interview for DSM-IV (SCID) to exclude individuals who meet the criteria for current psychoses, anxiety, or bipolar disorders, as well as the criteria for substance abuse; and (2) a 41-item questionnaire that asks for the presence of diabetes, hypertension, lungs, heart, kidney, or liver disease. The same questionnaire also asks for a history of head trauma, or other neurological disease, and also records the use of current medications, smoking (nicotine), alcohol, and caffeine, and whether they are currently receiving clinical treatment for obesity or following a certain diet. Subjects who meet a psychiatric diagnosis, or report a history of head injury or neurological disease, or current use of medications that impact the central nervous system, including nicotine, or are currently in clinical treatment for obesity and follow a certain diet are excluded. All participants and their parents (for participants under 18) gave informed consent to the study procedures, which were approved by the University of Southern California Institutional Review Board (reference number UP-10-00052).

**Table 1:** **Descriptive statistics of all behavior measures and dietary intake**

	Mean	SD	Range	Gender difference
**BMI (kg/m** ^**2**^ **)**	23.1	3.0	19.1-33.7	t = 1.67, p = .11
**IQ**	117.5	9.5	103-136	t = .85, p = .40
**SOPT**	65.1	3.5	56-70	t = .31, p = .76
**Hungry Rating**	2.6	2.0	1-4	t = .86, p = .40
**NDSR Low-Calorie Foods**	2.4	1.6	.1-7.5	t = 2.76, p < .01**
**NDSR High-Calorie Foods**	1.8	1.3	0-5.7	t = .64, p = .53

**p < .01. *SD* Standard Deviation, *BMI* Body mass index, *SOPT* Self-ordered pointing task, *NDSR* Nutrition data system for research.

### Procedures

**Figure 1:**
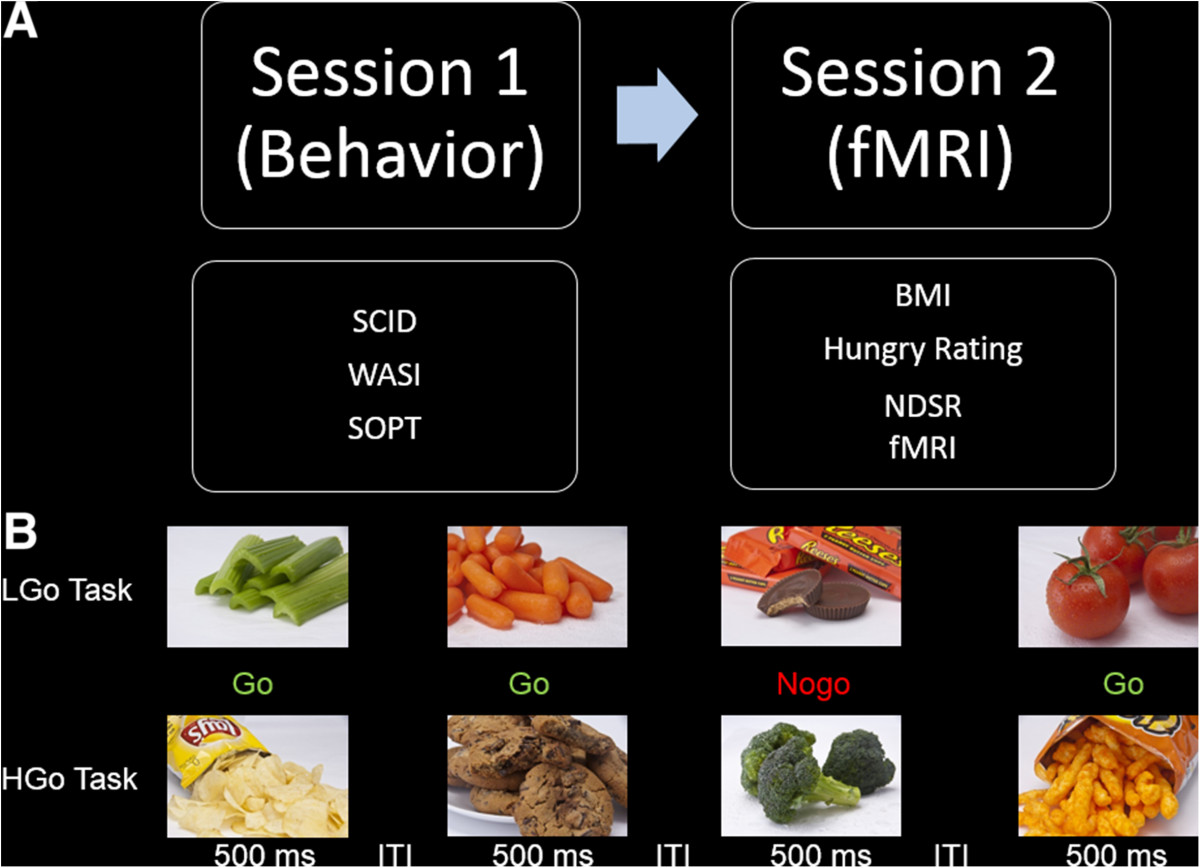
**Design of the study. A)** The schematic of the procedure. Participants were asked to visit the lab for two sessions: one behavior session and one fMRI session. **B)** The illustration of the event-related food-specific go/nogo tasks 1) low-calorie food go/high-calorie food nogo task (LGo task), and 2) high-calorie food go/low-calorie food nogo task (HGo task). Participants were asked to press a button as soon as possible to the go trials (vegetable pictures in LGo task and snack pictures in HGo task) and withhold the response to the nogo trials (snack pictures in LGo task and vegetable pictures in HGo task). The order of tasks was counterbalanced across subjects. SCID: structured clinical interview for DSM-IV; WASI: Wechsler abbreviated scale of intelligence; SOPT: self-ordered pointing task; BMI: body mass index; NDSR: nutrition data system for research; fMRI: functional magnetic resonance imaging; ITI: intertribal interval.

Participants were asked to come to the lab for two sessions (Figure 1A). During the first visit, participants (and a parent for those under 18) were asked to complete and sign the consent form(s), and complete the SCID and behavioral tasks. Participants were then scheduled to return for the fMRI scan session. Twenty-six subjects completed their scan in the morning between the hour of 10 am and 11 am, and only 4 subjects were scanned in the afternoon due to scheduling restraints. Participants were asked to refrain from any intense physical activity prior to scanning for 24 hours. Participants were asked to eat normally and to have their usual meal before they arrived for the fMRI session. Prior to the fMRI scan, height and weight were assessed using standard procedures, a 24-hour dietary recall was conducted, and participants rated their hunger level on a scale ranging from 1 (not hungry at all) to 10 (very hungry) to ensure that the participant was not in a deprived state. Subjects who provided a hunger rating > 5 were asked to reschedule their scan and to return after they had first consumed a normal meal. Thus all scanned participants rated their hunger as ≤ 4, with a mean score of 2.6.

### Measures

#### Behavior tasks

Participants were asked to complete two behavioral tasks (see Table 1). The Wechsler Abbreviated Scale of Intelligence WASI, [70] was used as an estimate for IQ, and the Self-Ordered Pointing Task SOPT, [71] was used as an index of working memory and executive functioning.

#### Dietary intake

A single, in-person 24-hour dietary recall was conducted by trained research staff using a multipass method facilitated by the Nutrition Data System for Research software NDSR, [72, 73]. All of the recalls were reported for a weekday. The software includes a dietary supplement assessment module so that nutrient intake from both food and supplemental sources may be captured and quantified. Based on the NDSR nutrient totals report, no individual was identified as reporting an implausible total energy intake (<500 or >7000 kcal) as suggested by a previous study [74]. Total daily servings of low-calorie foods and high-calorie foods were computed by summing total intake of fruits and vegetables (servings/day) and total intake of fatty foods and sugar-sweetened foods (servings/day), respectively. For the analyses, low and high-calorie food consumption (servings/day) were calorie-adjusted for total energy intake, as estimated by the dietary recall, and are reported as servings per 1000 kcals (servings/1000 kcals). The calorie-adjustment was performed to ensure that higher levels of intake were not artifacts of higher energy expenditure, as related to age or high levels of activity.

### fMRI tasks

Participants performed two food-specific go/nogo tasks in the scanner as follows: 1) a low-calorie food go and high-calorie food nogo task (LGo task), and 2) high-calorie food go and low-calorie food nogo task (HGo task). This go/nogo paradigm allows examination of the inhibition of prepotent responses to appetizing food items. Participants were asked to press a button as soon as possible to the go trials (low-calorie food pictures in LGo task and high-calorie food pictures in HGo task), and to withhold responses to the nogo trials (high-calorie food pictures in LGo/HNogo task and low-calorie food pictures in HGo/LNogo task). Examples of low-calorie food images included cucumbers, celery, broccoli, and carrots. Examples of high-calorie food images included chocolate bars, cookies, ice cream, and potato chips. All images of the foods observed are commonly available in everyday life (Figure 1B).

Each task consisted of 120 go trials (75%) and 40 nogo trials (25%). Nogo trials were presented in pseudo-randomized order, designed so that Nogo trials appeared with equal probability after 1 - 5 consecutive Go trial presentations, and no two Nogo trials appeared consecutively. Each stimulus was presented for 500 ms, followed by a fixation cross for 1.5 - 4 seconds with a mean of 2.5 s. The sequence was optimized for design efficiency using an in-house program. Each task ran for 8 minutes. The order of two versions of go-nogo tasks was counterbalanced across subjects.

Following signal detection theory, the hit rate, false alarm rate, sensitivity index d’ (*d*′ = *Z*_*hits rate*_ - *Z*_*false alarm rate*_) and decision bias C [*C* = - 0.5 × (*Z*_*hits rate*_ + *Z*_*false alarm rate*_)] were calculated for each task. The mean reaction time for go trials and nogo trials (false alarm trials only) for each task were also calculated. The reaction time for go trials served as an index for habitual responding to the stimuli, with longer reaction times indicating less habitual response; while decision bias C served as an index of response inhibition, with higher values indicating better inhibitory control.

### fMRI protocol

Prior to the scanning procedure, participants reviewed all stimuli used in the tasks and were informed by a research assistant of the category to which each stimulus belonged. During the fMRI scan, participants laid in the supine position on the scanner bed to view the task back-projected onto a screen through a mirror attached to the head coil. Foam pads were used to minimize head motion. Stimulus presentation and timing of all stimuli and response events were achieved using Matlab (Mathworks) and Psychtoolbox (http://www.psychtoolbox.org) on an IBM-compatible PC. Participants’ responses were collected online using an MRI-compatible button box.

fMRI imaging was conducted in a 3 T Siemens MAGNETOM Tim/Trio scanner in the Dana and David Dornsife Cognitive Neuroscience Imaging Center at the University of Southern California. Blood oxygen level dependent (BOLD) functional scanning used a z-shim gradient echo EPI (echo planer imaging) sequence with PACE (prospective acquisition correction). This specific sequence is dedicated to reduce signal loss in the prefrontal and orbitofrontal areas. The PACE option can help reduce the impact of head motion during data acquisition. The parameters are: TR/TE = 2000/25 ms; flip angle = 90°; 64 × 64 matrix size with resolution 3 × 3 mm^2^. Thirty-one 3.5-mm axial slices were used to cover the whole cerebral cortex and most of the cerebellum with no gap. The slices were tilted about 30° clockwise along the AC-PC plane to obtain better signals in the orbitofrontal cortex. The anatomical T1-weighted structural scan was done (TR/TE = 1950/2.26 ms; flip angle 7°; 176 sagittal slices; spatial resolution = 1 × 1 × 1.95 mm) for registration purpose.

### fMRI analysis

Image preprocessing and statistical analysis were carried out using FSL package (http://www.fmrib.ox.ac.uk/fsl). fMRI images were realigned to compensate for small residual head movements that were not captured by the PACE sequence [75]. Translational movement parameters never exceeded 1 voxel in any direction for any participant. Data were spatially smoothed using a 5-mm full-width-half-maximum (FWHM) Gaussian kernel. The data were filtered using a nonlinear high pass filter with a 100-second cut-off.

A two-step registration procedure was used whereby EPI images were first registered to the MPRAGE structural image, and then into standard MNI space, using affine transformations [75]. Registration from MPRAGE structural image to standard space was further refined using FNIRT nonlinear registration [76, 77]. Statistical analyses were performed in the native image space, with the statistical maps normalized to the standard space prior to higher-level analyses. The data were modeled at the first level using a general linear model (GLM) within FSL’s FILM module. Brain activation in every trial was modeled for go and nogo trials respectively in the single subject level. Error-related trials (misses and false alarms) were modeled together as a nuisance variable. The event onsets were convolved with the canonical hemodynamic response function (HRF, double-gamma) to generate regressors used in the GLM. Temporal derivatives were included as covariates of no interest to improve statistical sensitivity. Null events were not explicitly modeled, and therefore constituted an implicit baseline. The six movement parameters were also included as covariates in the model.

Higher-level analyses created cross-run contrasts for each subject using a fixed effects model. A 2 Task (Go vs Nogo) × 2 Stimuli (Low-calorie vs High-calorie food cues) within-subject factor design was used. Higher level random-effects models were tested for group analyses using FMRIB’s Local Analysis of Mixed Effect stage 1 only [78, 79] with automatic outlier detection [80]. First, a full 2 × 2 factor analysis was conducted at the group level to test for the main effects of task and stimuli as well as their interaction. Then, of particular interest to us, two additional hypotheses were evaluated at the group level as follows: (1) that high-calorie food nogo trials would elicit more activity in the prefrontal system than high-calorie food go trials; and (2) that high-calorie food go trials would elicit more activity in the habitual system than low-calorie food go trials. To rule out the possibility that the first contrast between nogo trials and go trials could partially be due to pre-potent go responses, a supplemental analysis was conducted by matching the number of go trials to the number of nogo trials. In this analysis, only the last go trial just prior to each nogo trial was included as an instance of the go trials. All other go trials were included as nuisance variables. This analysis generated similar findings. In all analyses, age and gender were included as covariates. Group images were evaluated with a height threshold of Z > 2.3 and a cluster probability of p < 0.05, corrected for whole-brain multiple comparisons based on Gaussian random field theory.

To correlate the nutrition data with brain activity, region of interests (ROI) were created from clusters of voxels with significant activation clusters in the voxel-wise analyses. Analyses were performed by extracting parameter estimates (betas) of each event type from the fitted model and averaging across all voxels in the cluster for each participant/session. Percent signal changes were calculated using a method suggested by Mumford (http://mumford.fmripower.org/perchange_guide.pdf). Robust regression was used to minimize the impact of outliers in the behavioral data, using iteratively reweighted least squares implemented in the *robustfit* command in the MATLAB Statistics Toolbox [81]. Reported r-values reflect (non-robust) Pearson product-moment correlation values, whereas the reported p-values and regression lines are based on the robust regression results [81]. This correlational analysis is highly relevant to determining whether increased activity in the prefrontal system on the high-calorie food nogo trials is due to simply an inhibition of a pre-potent response, or a greater difficulty in inhibiting a response to high-calorie foods.

## Results

### Behavioral tasks and dietary intake

Table 1 shows the mean, standard deviation, and range of the measures collected from the behavioral tasks and the 24-hour diet recall. Our results revealed normal intelligence (IQ) and working memory/executive functioning in our participants. BMI did not correlate with either IQ (*r = -.04, p = .83*) or working memory capacity (*r = -.15, p = .46*). Also, BMI did not correlate with age (*r = -.13, p = .50*) or differ between genders (*t = 1.67, p = .11*). With respect to dietary intake, participants reported consuming 2.4 ± 1.6 servings/day/1ooo kcals of low-calorie foods (i.e., fruits and vegetables) and 1.8 ± 1.3 servings/day/1000 kcals of high-calorie foods (i.e., fatty foods and sugar-sweetened foods). Subjects showed a trend of consuming more servings/day/1000 kcals of low-calorie foods than high-calorie foods (*t(29) = 1.70, p = .10*). The consumption of high-calorie foods (servings/day/1000 kcals) was independent of age (*r = .04, p = .84*), BMI (*r = -.04, p = .84*), and hunger rating (*r = -.15, p = .48*). There were no gender differences with respect to IQ, SOPT scores, hunger rating, or consumption of high-calorie foods per 1000 kcal (Table 1). However, there was a significant gender difference in consumption of low-calorie foods per 1000 kcal (*t*(28) = 2.76, *p* < .01), with females reporting more consumption of low-calorie foods per 1000 kcal (3.0 ± 1.6) than males (1.6 ± 1.1).

### Behavioral results in fMRI tasks

Table 2 summarizes the major behavioral measures for the fMRI go/nogo tasks, including hit rates, false alarm rates, sensitivity index d’, decision bias C, and reaction times for go trials and nogo trials (false alarm rates or inhibitory failures only). For each behavioral measure, paired t-tests were performed to test the difference between tasks (LGo vs HGo task). Analyses revealed that the false alarm rate in the LGo task was higher than in the HGo task (*t* = 3.03, *p* < .01). Similarly, decision bias C in the LGo task was smaller than in the HGo task (*t* = -4.05, *p* < .01). Both effects survived the Bonferroni correction for multiple comparisons. These results suggest that participants made more errors and had a harder time inhibiting responses to high-calorie food cues in the LGo task. Results also suggest that the reaction time for go trials tended to be longer in the LGo task than in the HGo task (*t* = 1.91, *p* = .06), although this effect did not survive the Bonferroni correction for multiple comparisons. This suggests that participants tended to more readily press the response button when go trials consisted of high-calorie food images in the HGo task. No other significant differences were found (all *p* > .05).

**Table 2:** **Behavioral measures from the food-specific go/nogo task**

	LGo task	HGo task	t statistics
**Hits Rate**	.92 ± .07	.91 ± .06	t = 1.52, p = .14
**False Alarm Rate**	.18 ± .11	.13 ± .09	t = 3.03, p = .005**
**Go trial RT (ms)**	501.6 ± 73.7	484.2 ± 65.2	t = -1.91, p = .06
**Nogo trial RT (ms)**	433.1 ± 80.1	419.4 ± 80.7	t = -.80, p = .43
**d’**	2.52 ± .49	2.58 ± .58	t = -.64, p = .53
**C**	-.34 ± .42	-.10 ± .39	t = -4.05, p < .001**

**p < .01 corrected for multiple comparison using Bonferroni correction. *LGo Task* low-calorie food go/high-calorie food nogo task, *HGo Task* high-calorie food go/low-calorie food nogo task, *RT* reaction time.

Finally, controlling for participants’ age and gender, several correlations were significant among the behavioral measures. Reaction time for the go trials in the HGo task was negatively correlated with both BMI (*r* = -.60, *p* < .01) and high-calorie food consumption (*r* = -.50, *p* < .05), suggesting the habitual response to the high-calorie foods was greater for individuals with higher BMI, and individuals who reportedly consumed more high-calorie foods. The decision bias C for the LGo task negatively correlated with both BMI (*r* = -.47, *p* < .05) and high-calorie food consumption (*r* = -.49, *p* < .05), suggesting the inhibiting response to the high-calorie foods was more difficult for individuals with higher BMI and individuals who reported consuming more high-calorie food.

### fMRI data

**Figure 2:**
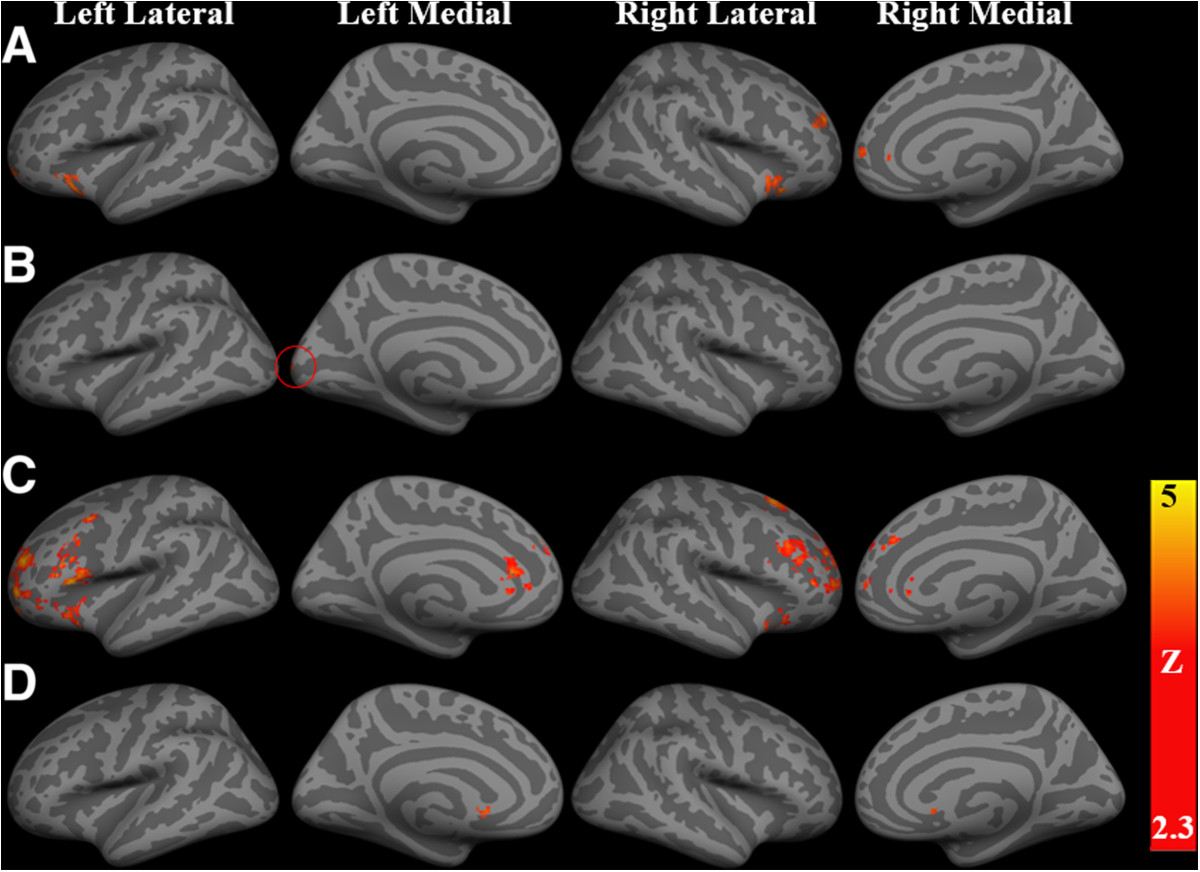
**Summary of fMRI results rendered onto an averaged brain by FreeSurfer. A)** Several brain regions showed a significant main effect of Task, including bilateral frontal pole, bilateral dorsolateral prefrontal cortical (DLPFC) extending to insular cortex, and anterior cingulate cortex (ACC). Nogo trials activated more than go trials in these regions. **B)** The left occipital pole (circled) showed a significant main effect of Stimuli with high-calorie food activated more than low-calorie food. **C)** The prefrontal system showed higher activation for high-calorie food nogo trials than high-calorie food go trials, including bilateral DLPFC, insula, frontal pole, ACC and right superior frontal gyrus. **D)** The "habitual" system (right striatum) showed higher activity in high-calorie food go trials relative to low-calorie food go trials. No brain region showed an interaction effect between Task and Stimuli.

Table 3 summarized the fMRI results. First, imaging results suggested a significant main effect of Task in a few brain regions, including bilateral frontal pole, bilateral dorsolateral prefrontal cortex (DLPFC) extending to the insular cortex, and anterior cingulate cortex (ACC). These brain regions were activated more during nogo trials than during go trials (Table 3 and Figure 2A). Second, the left occipital pole showed a significant main effect of stimuli with the high-calorie food pictures activating this region more than the low-calorie food pictures (Table 3 and Figure 2B). Third, no interaction effect between task and stimuli was found in any brain region.

**Table 3:** **Summary of the fMRI results (factor analysis)**

Hemisphere	Brain region	Voxels	x	y	z	Z
Main Effect of Task (Nogo > Go)						
L	DLPFC/Insula	280	-32	24	-8	3.56
R	Frontal Pole	221	28	46	26	3.42
R	DLPFC/Insula	218	32	26	-14	3.12
L	Frontal Pole	99	-22	58	-6	3.25
L/R	ACC	92	4	44	4	2.85
**Main Effect of Stimuli (High-calorie > Low-calorie Food Cues)**						
L	Occipital Pole	182	-6	-100	4	3.28
**Interaction between Task and Stimuli**						
None						

*L* Left, *R* Right, *ACC* Anterior Cingulate Cortex, *DLPFC* Dorsolateral Prefrontal Cortex.

**Figure 3:**
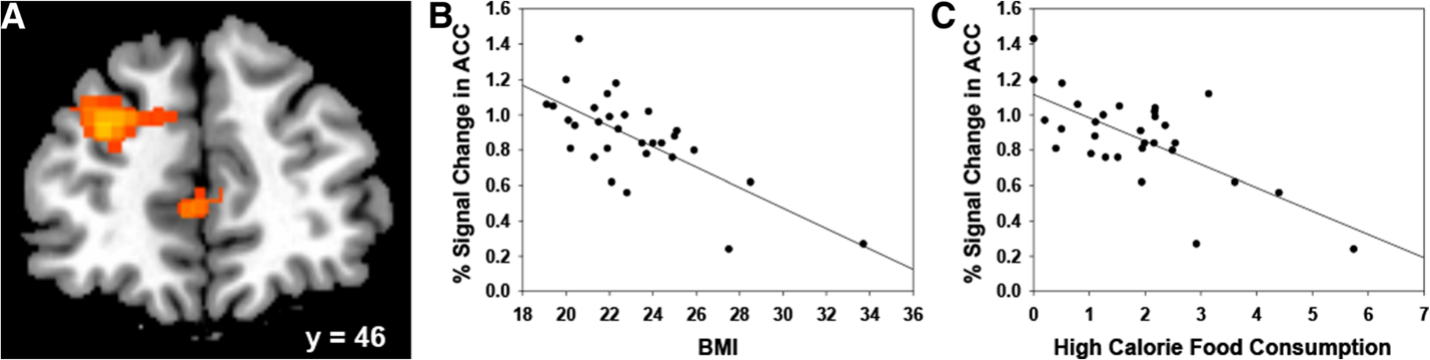
**Activity of the ACC was inversely correlated with both BMI and NDSR. A)** The ACC was activated when comparing nogo trials to go trials. Slices are displayed in radiological view (right is on the viewer’s left). **B)** Scatter plot showed the correlation between ACC activation and BMI. **C)** Scatter plot showed the correlation between ACC activation and high-calorie food consumption tested by NDSR. ACC: anterior cingulate cortex; BMI: body mass index; NDSR: nutrition data system for research.

Analyses comparing the high-calorie food go and nogo trials revealed that the neural systems referred to as the "reflective" system or prefrontal system [29, 82] showed more activation during nogo trials than go trials, including bilateral activation of the DLPFC, insula, frontal pole, and ACC, and activation of the right superior frontal gyrus (Table 4 and Figure 2C). Supplemental analysis showed similar results when matching the number of go trials to the number of nogo trials. Further, of particular interest to us, ROI analysis suggested that the activation in the ACC region when comparing nogo trials to go trials (Figure 3A, MNI = 4, 44, 4) was negatively correlated with BMI (Figure 3B; *r* = -.71, *p* < .01) and high-calorie food consumption as measured by the 24-hour recall/NDSR (Figure 3C; *r* = -.69, *p* < .01). Also, females showed a trend of more activation in ACC than males when comparing nogo trials to go trials (*t* = 1.97, *p* = .06).

**Table 4:** **fMRI results related to habitual and prefrontal system**

Hemisphere	Brain region	Voxels	x	y	z	Z
Nogo > Go (High-calorie Food Cues Only)						
L/R	ACC extending to left Frontal Pole	1458	-30	50	2	3.50
L	DLPFC/Insula	1220	-40	20	8	3.73
R	DLPFC	840	44	18	26	3.24
L	Frontal Pole	308	-24	62	-6	3.76
R	Frontal Pole	214	34	42	4	3.47
R	Superior Frontal Gyrus	205	18	22	58	3.56
R	Insula/Orbital Frontal Cortex	185	36	28	-22	2.93
**High-calorie > Low-calorie Food Cues (Go Trials Only)**						
R	Striatum	60	10	12	2	2.74

*L* Left, *R* Right, *ACC* Anterior Cingulate Cortex, *DLPFC* Dorsolateral Prefrontal Cortex.

**Figure 4:**
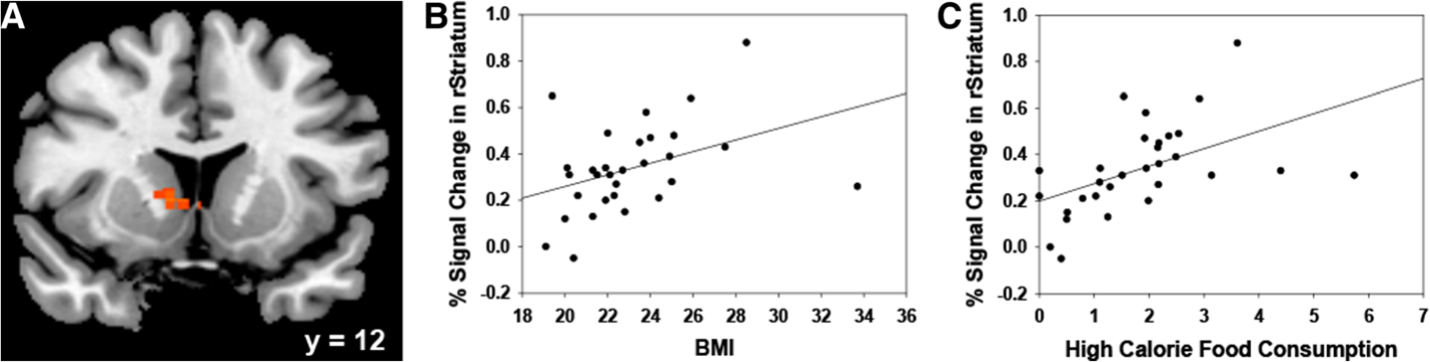
**Activity of the striatum was positively correlated with both BMI and NDSR. A)** The activation of the right striatum was revealed when comparing snack go trials to vegetable go trials. Slices are displayed in radiological view (right is on the viewer’s left). **B)** Scatter plot showed the correlation between the right striatum activation and BMI. **C)** Scatter plot showed the correlation between the right striatum activation and high-calorie food consumption tested by NDSR. BMI: body mass index; NDSR: nutrition data system for research.

Results comparing go trials only revealed that the high-calorie food cues were associated with higher activity in the right striatum relative to low-calorie food cues (Table 4 and Figure 2D), a region belonging to what we have referred to as the "habitual" system. ROI analysis suggested that the degree of this increased activity in the right striatum (Figure 4A; MNI = 10, 12, 2) was positively correlated with both BMI (Figure 4B; *r* = .39, *p* < .05) and level of actual high-calorie food consumption in real-life, assessed with the NDSR (Figure 4C; *r* = .50, *p* < .01). There was no significant difference between males and females in the activation of right striatum (*t* = .49, *p* = .63).

## Discussion

The present study used varieties of food specific go/nogo tasks to investigate the neural activity underlying response inhibition or the ability to control one’s impulses when faced with images of high-calorie foods. Behaviorally, participants more readily pressed a response button when go trials signaled high-calorie foods during a high-calorie food go and low-calorie food nogo task (HGo task). We interpret these results to mean that the high-calorie food stimuli elicited more habitual responding and this response to high-calorie food stimuli was significantly greater for individuals with higher BMI, and also for those who consumed more high-calorie foods. Furthermore, it was more difficult for participants to inhibit their response to high-calorie foods in the LGo task (low-calorie food go and high-calorie food nogo) than low-calorie foods in the HGo task. Similar to habitual responding to high-calorie foods, the ability to inhibit responding to high-calorie foods was more difficult for individuals with higher BMI and individuals who reported consuming more high-calorie foods. The fMRI findings further supported our hypotheses that (1) the habitual system was activated more in response to high-calorie food go trials than low-calorie food go trials, and its activation correlated with participants’ BMI as well as their consumption of high-calorie foods; and (2) the prefrontal system was more engaged during nogo trials than the go trials, and its activation was inversely correlated with BMI and high-calorie food consumption.

The habitual system observed in the present study was located in the right striatum, which is a dopamine-dependent neural system critical for the incentive motivational effects of a variety of non-natural rewards (e.g., psychoactive drugs) and natural rewards (e.g., food) [32, 83–89]. This is also the neural system that has been argued to be responsible for the transfer of reward seeking from controlled to automatic and habitual behaviors [32]. The so-called "habitual" system has been shown to become hyperactive and to exaggerate the incentive value of rewards in individuals with substance abuse problems [28]. Given the similarities between the responses to images of high-calorie foods we observed in this study and to those responses others have observed among substance (ab)users to drug-related cues, this study adds to the evidence supporting a food-addiction model of obesity. Further, our findings are consistent with previous studies that have demonstrated that food cues (such as the sight or smell of food) engage the mesolimbic dopamine-striatal system, especially in obese individuals [13, 16, 46–52, 90, 91]. For example, emerging findings suggest that the fat and sugar present in high-calorie foods may particularly engage these dopamine-dependent reward systems [13] and motivate food seeking behaviors [16, 46, 61]. fMRI studies also show that compared to non-food-related pictures, food-related pictures activate the striatum [92] in healthy individuals. Consistent with this finding, we observed right striatum activation when responses to high-calorie food pictures were compared to responses to low-calorie food pictures, although previous studies showed that the dorsal striatum is not strictly dedicated to habit behaviors, and that it can be involved in decision-making [28, 82, 93–95]. Animal studies also have shown that direct pharmacological activation of the striatum, amygdalo-hypothalamic circuit produced hyperphagia and increased preferentially the intake of foods high in fat and sugar, even in animals fed beyond apparent satiety [96].

Additionally, we found that activation of the "habitual" system correlated with both BMI and daily consumption of high-calorie foods. However, we did not find a significant correlation between BMI and high-calorie food consumption behaviorally. The present study consisted of a sample of mostly normal-weight participants, and this lack of variance in BMI might preclude replicating behavioral findings observed in other studies between obese and normal weight individuals in the consumption of high-calorie foods [92, 97]. Nevertheless, the significant correlation between brain activation and BMI, as well as daily consumption of high-calorie foods, is consistent with several lines of evidence that suggest that highly palatable food may induce greater incentive values in obese individuals compared to normal controls [92, 97]. Behavioral studies also show that overweight children indicate that high-calorie food consumption (e.g., snack foods) is more reinforcing than what their leaner peers indicate [97]. The relative reinforcing value of food versus two non-food alternatives (e.g., time spent playing a hand-held video game or time spent reading magazines or completing word searches or mazes) is also high in overweight children, and relatively low in non-overweight children [97]. Using fMRI, Beaver and colleagues [92] reported that individual variation in trait reward sensitivity correlated highly with activation to images of highly palatable, appetizing foods (e.g., chocolate, ice cream) in a fronto-striatal-amygdala-midbrain network in healthy volunteers. Beaver and colleagues’ study, together with our findings in adolescents and young adults, suggest that the habitual system may be hyper-active when facing highly palatable and high-calorie foods, such that it begins to exaggerate the incentive impact of food reward, contributing to overweight and obesity.

This study further showed that the nogo trials relative go trials engaged several regions implicated in the prefrontal system, including the ACC and bilateral frontal pole, when participants performed the food-specific go/nogo task. Implicated regions of the prefrontal system including the ACC, frontal poles and DLPFC help to control basic impulses and allow more flexible pursuit of long-term goals [98, 99]. The prefrontal system is important for good decision-making and inhibitory control. Several studies suggest that the cognitive or regulatory control of food intake is in the prefrontal system [100–103], and this system shows an altered response to food cues [59, 60]. For example, one study showed that lean individuals had greater neural activity than obese individuals in the prefrontal cortex when inhibiting food consumption due to satiation [101]. Consistent with our findings, another study found significant increases in the activation of the OFC in response to high-calorie food images, but not to low-calorie food images, suggesting change in reward evaluation and response inhibition in reaction to fatty and unhealthy food images [102]. Further, the prefrontal system appears to be hypo-active for those who consume more high-calorie food daily as reflected by the inverse correlation between ACC activation and high-calorie food consumption.

Gender differences in decision-making and inhibitory control processes have been observed with respect to various appetitive/addictive behaviors [104–113]. For example, males have been found to be more likely than females to engage in risky behaviors [110], and males appear to be less able to control inappropriate behaviors than females [111]. One study found that after consumption of the same amount of alcohol, the capacity to inhibit or control behavior was less impaired in women relative to men [112]. Another study found marked gender differences in inhibitory control with respect to consumption of sugar-sweetened snacks on food-cued and generic go/nogo tasks [113]. Inhibitory problems measured with these tasks were strong correlates of sweet snack consumption in males, but not in females. Similar to these findings, the present study found that females consumed more low-calorie food than males, which might be related to the notion that they have a stronger neural system for impulse control towards high-calorie food, and this is supported by the finding of higher ACC activation in the food-specific go/nogo task.

The present study has several limitations to be addressed in future studies. First, we did not find a correlation between BMI and high-calorie food consumption. This may simply be due to measurement error that is inherent in a single dietary assessment or possibly due to variations in developmental status (e.g., continued maturation, particularly among the young men) [114–118] or physical activity energy expenditure in this young adult population. Second, participants were provided instructions for eating prior to the study session. Their fed state was confirmed using self-reported, perceived hunger status rather than providing a standardized meal before the scan. Although providing a standardized meal could minimize the influence of hunger status on task performance [119, 120], it could also introduce a confounding factor; that is, "liking" or "disliking" the meal. Examining potential differences in neural responses due to eating standardized versus normal meals prior to scanning should be conducted in future studies. Third, we did not measure levels of appetite hormones prior to scanning in this study. Again, this would be an important question to address in future studies given the available evidence showing that these hormones are important to decision making around diet and nutrition [121]. Another important aspect of inhibitory control not addressed in the current study is the role of cognitive restraint that participants exert over eating behavior. Previous studies have shown that the cognitive restraint participants exert over eating behavior influences brain activity during an inhibitory task [122, 123]. Though we did not explicitly assess cognitive dietary restraint or weight control status in this sample, it is an important issue that deserves attention in future research. Finally, our subjects did not consume real food while in the scanner; instead, they viewed images of food. This design could potentially reduce the ecological validity of the task, although numerous fMRI studies conducted to date have used food images instead of real food to measure food choice [11, 17, 18, 47, 92, 124–126].

## Conclusion

In conclusion, the present study among adolescents and young adults used food-specific go/nogo tasks to investigate brain activity underlying self-control when faced with tempting food choices. Results confirmed our hypotheses regarding two key neural systems involved in decisions to seek reward from appetizing foods: a hyper-active habitual system and a hypo-active prefrontal system in this population. Though the cross-sectional design does not permit inferences regarding whether the inhibitory control deficits and hyper-responsivity of reward regions are individual vulnerability factors for overeating or results of habitual overeating. Our results shed light on the neural basis of one’s loss of ability to self-control when faced with tempting food choices, which could potentially contribute to the development of intervention strategies aimed at reducing the consumption of high-calorie foods. Such intervention strategies are important in order to help reduce the incidence of obesity as adolescents and young adults approach adulthood, thereby reducing the future risk of obesity-related chronic diseases and cancers.

## Authors’ original submitted files for images

Below are the links to the authors’ original submitted files for images. Authors’ original file for figure 1 Authors’ original file for figure 2 Authors’ original file for figure 3 Authors’ original file for figure 4
