# Supplementary figures and images for: Poor ability to resist tempting calorie rich food is linked to altered balance between neural systems involved in urge and self-control
Source: Nutr J. 2014 Sep 16;13:92. doi: 10.1186/1475-2891-13-92 (PMC4172871; doi:10.1186/1475-2891-13-92)

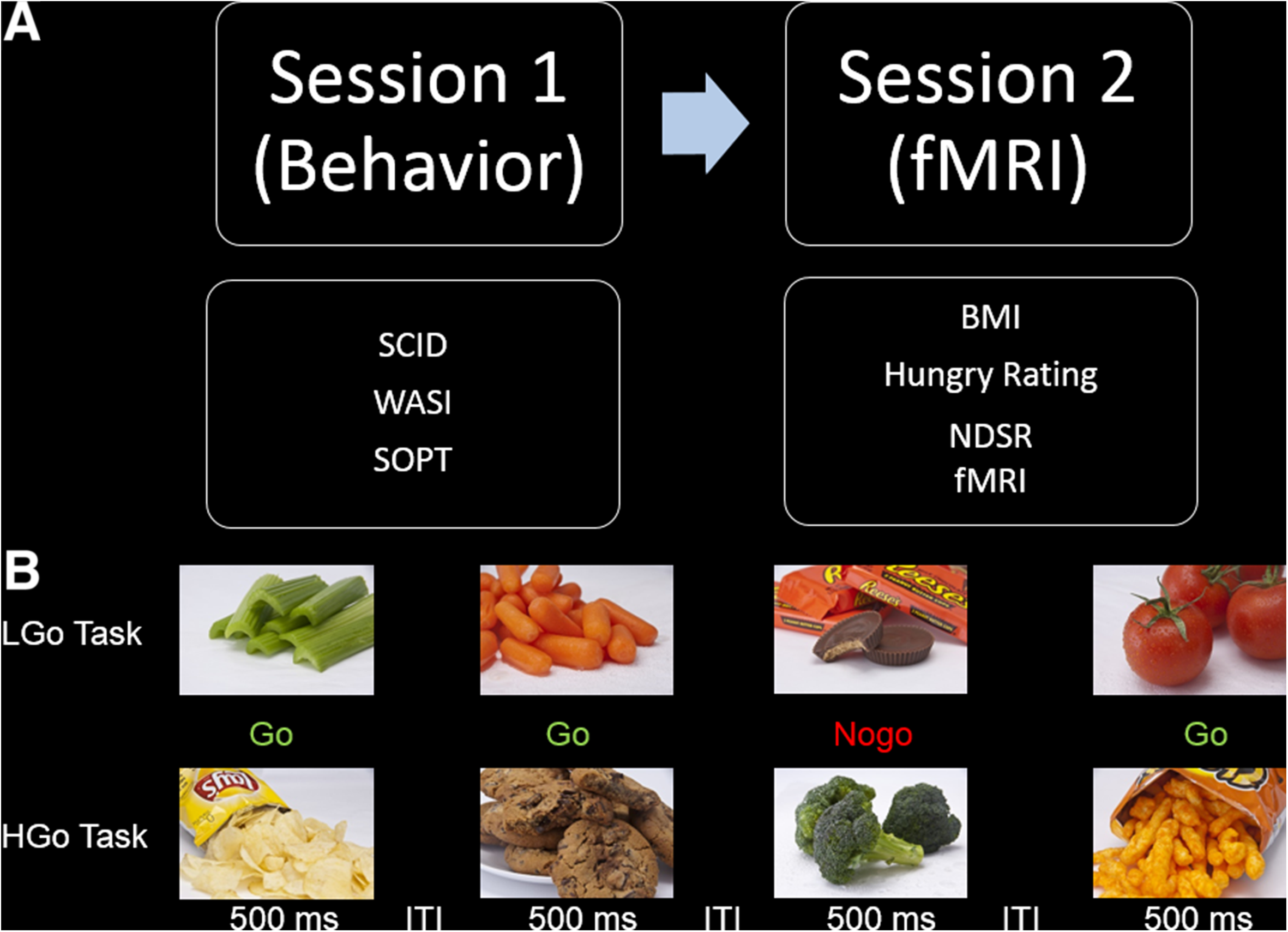

Supplement: Supplementary file 1 — Authors’ original file for figure 1 [file 12937_2014_828_MOESM1_ESM.tif]

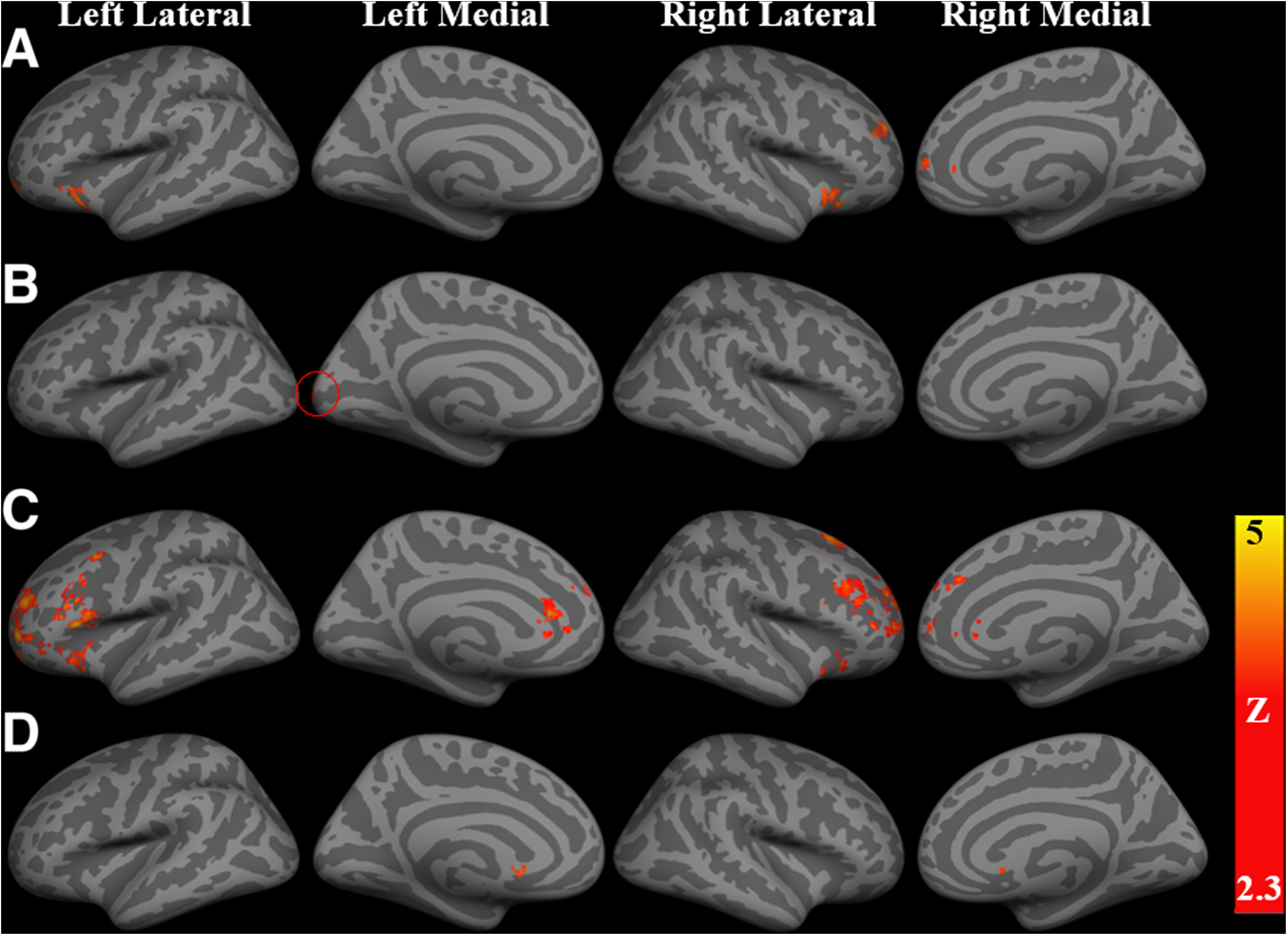

Supplement: Supplementary file 2 — Authors’ original file for figure 2 [file 12937_2014_828_MOESM2_ESM.tif]

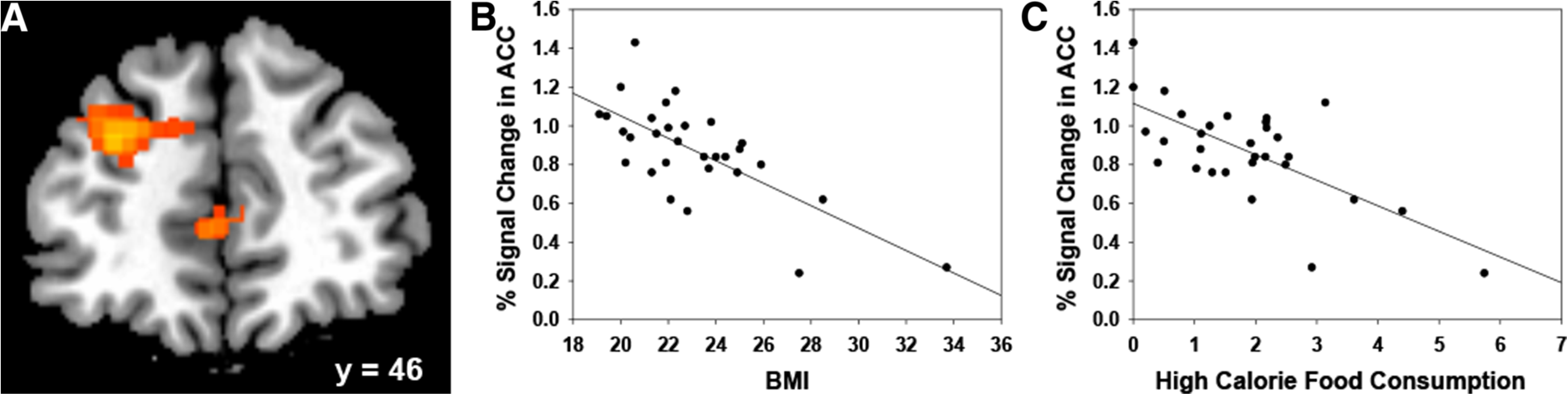

Supplement: Supplementary file 3 — Authors’ original file for figure 3 [file 12937_2014_828_MOESM3_ESM.tif]

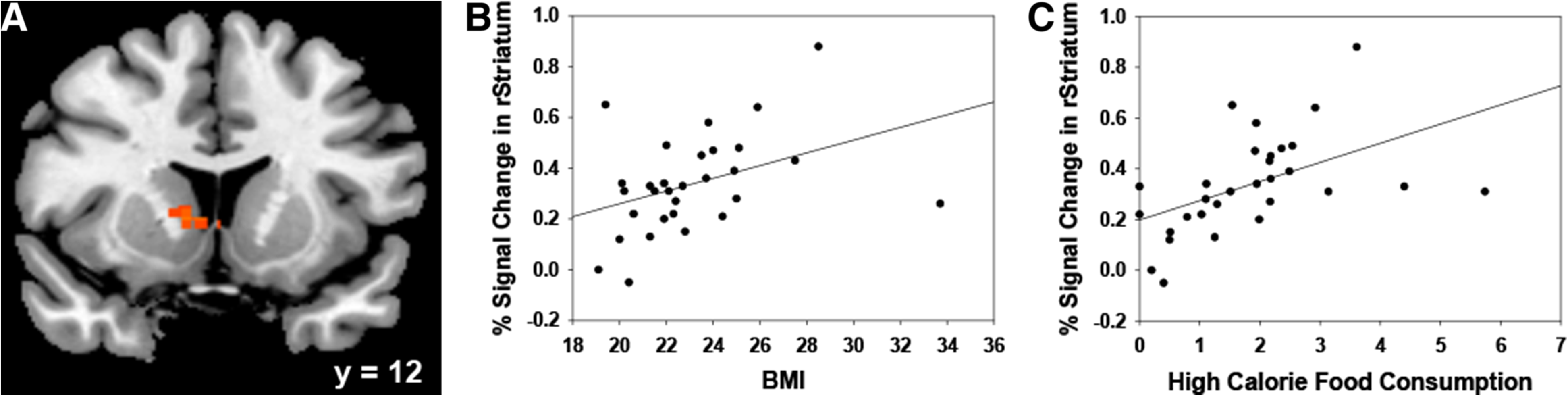

Supplement: Supplementary file 4 — Authors’ original file for figure 4 [file 12937_2014_828_MOESM4_ESM.tif]
